# In Situ Observation of Thermoelastic Martensitic Transformation of Cu-Al-Mn Cryogenic Shape Memory Alloy with Compressive Stress

**DOI:** 10.3390/ma15113794

**Published:** 2022-05-26

**Authors:** Zhenyu Bian, Jian Song, Pingping Liu, Farong Wan, Yu Lei, Qicong Wang, Shanwu Yang, Qian Zhan, Liubiao Chen, Junjie Wang

**Affiliations:** 1 School of Materials Science and Engineering, University of Science and Technology Beijing, Beijing 100083, China; bianzhenyu@c.ccs.org.cn (Z.B.); zybian@ccs.org.cn (J.S.); qwea142311@163.com (Q.W.); yangsw@mater.ustb.edu.cn (S.Y.); qzhan@mater.ustb.edu.cn (Q.Z.); 2 China Classification Society Certification Co., Ltd., Beijing 100006, China; 3 Faculty of Engineering, Hokkaido University, Sapporo 060-8628, Japan; 18610132651@163.com; 4 Key Laboratory of Cryogenics, Technical Institute of Physics and Chemistry, Chinese Academy of Sciences, Beijing 100190, China; chenliubiao@mail.ipc.ac.cn (L.C.); wangjunjie@mail.ipc.ac.cn (J.W.)

**Keywords:** cryogenic shape memory alloys, compressive stress, cryogenic metallography, in situ observation, residual austenite, synchrotron radiation

## Abstract

The thermoelastic martensitic transformation and its reverse transformation of the Cu-Al-Mn cryogenic shape memory alloy, both with and without compressive stress, has been dynamically in situ observed. During the process of thermoelastic martensitic transformation, martensite nucleates and gradually grow up as they cool, and shrink to disappearance as they heat. The order of martensite disappearance is just opposite to that of their formation. Observations of the self-accommodation of martensite variants, which were carried out by using a low temperature metallographic in situ observation apparatus, showed that the variants could interact with each other. The results of in situ synchrotron radiation X-ray and metallographic observation also suggested there were some residual austenites, even if the temperature was below M_f_, which means the martensitic transformation could not be 100% accomplished. The external compressive stress would promote the preferential formation of martensite with some orientation, and also hinder the formation of martensite with other nonequivalent directions. The possible mechanism of the martensitic reverse transformation is discussed.

## 1. Introduction

Thermoelastic martensitic transformation and its reverse transformation are the root of the shape memory effect (SME) and a unique part of martensitic transformation research. Shape memory alloy (SMA) based on SME has been widely used in many areas. Cu-based SMA, for example, has been widely used in intelligent valves, connectors, dampers and seismic attenuation due to its SME and high damping properties [1,2,3]. It also has the great potential of being used in cryogenic sealing because of its pseudoelasticity, which is also based on the thermoelastic martensite [4,5,6].

SME is now generally well understood and generally considered to be associated with the reversibility of the martensitic transformation, that is, the thermoelastic martensitic transformation. As early as 1938, Greninger and Mooradian first discovered the phenomenon of martensite growth and disappearance with temperature fall and rise in the Cu-Zn alloy during their investigation of copper alloys (copper–zinc and copper–tin) [7]. Then, in about 1949, Kurdjumov and Khandros named this reversible transformation as “thermoelastic martensitic transformation” after a detailed study of thermoelasticity in Cu-Zn and Cu-Al-Ni alloys [1,8].

Many aspects of thermoelastic martensitic transformation have been studied in detail [9], including the associated crystallographic behavior [10], the thermodynamics [11,12], the effects of chemical composition [13,14,15,16] and post-heat treatment [3,17,18,19,20,21,22]. A notable phenomenon in the thermoelastic martensitic transformation of SMA is that an austenite phase grain can transform to different martensitic variants during the martensitic transformation [23], but all the martensite variants have to transform to the same original austenite phase grain instead of forming different austenite phase grains during the revise transformation. This phenomenon is very important because it is the basis of SME. Otsuka and Shimizu [24] discussed the effects of ordering on the crystallographic reversibility of the martensitic transformation and concluded that the complete reversibility of the martensitic transformation is characteristic of ordered alloys. However, they also note that the fcc-to-fct (face-centered-tetragonal) transformation is an “exception”. Bhattacharya et al. [25] provide an explanation for the reversibility on the basis of the symmetry change during the transformation. They show, through rigorous mathematical theory and numerical simulation, that irreversibility is inevitable in a “reconstructive” phase transformation, but not in a “weak” martensitic transformation, in which the symmetry group of both the parent and product phases are included in a common finite symmetry group (which includes symmetry breaking). Our previous work shows that there are always some residual austenites, even if the temperature is lower than M_f_ and the last formed martensite on cooling firstly disappeared on heating, through a low temperature metallographic in situ observation in a Cu-Al-Mn alloy [26]. However, so far, there is still much work remaining to understanding the effects of external compressive stress on the process of martensitic transformation and its reverse transformation. The direct observation and detailed knowledge of the reverse thermoelastic martensitic transformation of SMA are essential for the mechanism of thermoelastic martensitic transformation and the proper understanding of SME.

In this paper, a Cu-Al-Mn cryogenic SMA (martensitic transformation start temperature, Ms, is as low as about 100 K) was prepared. The thermoelastic martensitic transformation and its reverse transformation of the alloy have been observed in situ with and without compressive stress. The possible mechanism of martensitic reverse transformation is also discussed.

## 2. Experimental Procedure

**Figure 1:**
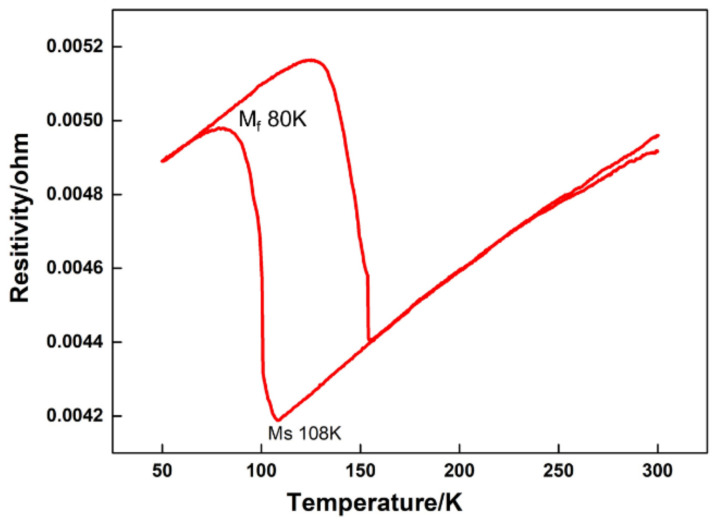
The Ms temperature of Cu-Al-Mn shape memory alloys.

The material analyzed in this study was a Cu-Al-Mn cryogenic SMA. The chemical composition and Ms point are listed in Table 1. The Cu-Al-Mn alloy was melted in a vacuum induction furnace with high purity Cu, Al and Mn [26]. The bulk specimen with a size of 20 mm × 15 mm × 5 mm was cut from the sample by wire cutting, and then heat treated by water quenching after holding at 900 °C for 10 min. The Ms measurement was performed using a PPMS-9 (Quantum Design, China). The Ms and martensitic transformation finish temperature (M_f_) are shown in Figure 1. Ms is about 108 K and M_f_ is about 80 K.

**Figure 2:**
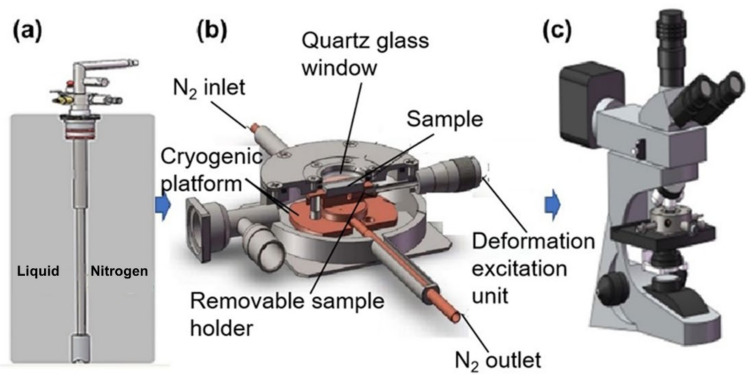
In situ observation apparatus of cryogenic metallographic with deformation excitation unit. (**a**) the pipe connecting the sample table and the liquid nitrogen tank, through which liquid nitrogen is introduced into the sample stage (**b**) sample stage with deformation excitation unit and (**c**) the metallographic microscope is equipped with micro image processing system (MIPs).

**Figure 3:**
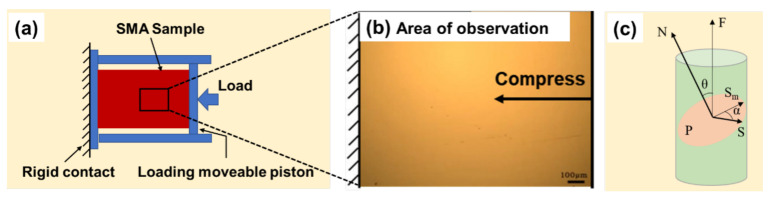
A schematic diagram of the area of observation with external stress. (**a**) Schematic diagram of observation window of copper sample table; (**b**) the picture of area of observation and (**c**) schematic diagram of compressive stress.

The working schematic diagram of the low temperature metallographic in situ observation instrument with deformation excitation unit is shown in Figure 2. The deformation-adjusting device on one side of the instrument can apply compressive stress to the sample, as shown in Figure 3. The schematic diagram is shown in Figure 3a–c. The sample was an ordinary metallographic one with maximum size of 20 mm × 20 mm × 8 mm. Cooling medium was liquid nitrogen. The temperature control accuracy was ±2 K, the minimum temperature could be reduced to 77 K, and the cooling rate was 45 K/min. The phase transformation process of the Cu-Al-Mn cryogenic SMA with and without compress stress could be observed through the quartz glass window of this instrument. In situ XRD experiments were carried out at the beamline 4B9A in Beijing Synchrotron Radiation Facilities (BSRF). Two XRD patterns were, respectively, collected at 293 K and 77 K under low-vacuum conditions (0.1 Pa) with an incident X-ray wavelength of 1.54 Å. The data collection time for the low temperature XRD pattern was about 2 h.

## 3. Experimental Results

### 3.1. Thermoelastic Martensitic Transformation and Reverse Transformation without Compressive Stress

The cryogenic metallographic images of the surface of the Cu-Al-Mn alloy with the change of the temperature are shown in Figure 4. The martensitic transformation process during cooling is shown in images 1–5 in Figure 4, and the reverse martensitic transformation process during heating is shown in images 6–10 in Figure 4; no loading pressure was applied to the sample when cooling and heating.

**Figure 4:**
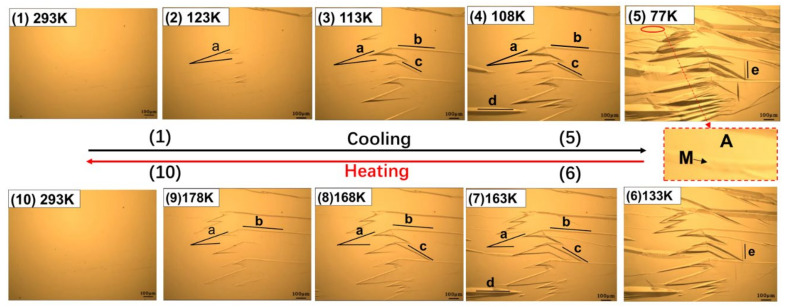
In situ observation of the thermoelastic martensitic transformation (1–5) and reverse transformation (6–10) without compressive stress. ‘A’ is austenite and ‘M’ is martensite.

The original sample surface is smooth, as shown in image Figure 4(1), which is at the fully complete β1 phase at 293 K. As the temperature decreases, the martensite variants with mark of “a”, “b”, “c”, “d” and “e” gradually appear on the sample surface, in the order of “a” → “b” → “c” → “d” → “e”, as shown in the images 2–5 of Figure 4. The martensites with different orientations grow continuously in their length and width directions. These preferentially grown martensite strips can interact with other oriented martensites subsequently formed. When the temperature was reduced to 77 K, and was held for a while, it was shown that there were still a few regions which remained in their original austenite phases and would not translate into martensites, as indicated by a ellipse mark in image 5 of Figure 4, and in the larger version of this figure.

As shown in images 6–10 of Figure 4, the martensitic variants of five orientations faded away and the sample surface returned to its original smoothness once again. The order of martensite disappearance is “e” → “d” → “c” → “b” → “a”, which is just opposite to the order of their formation.

### 3.2. Thermoelastic Martensitic Transformation and Reverse Transformation with Compressive Stress

**Figure 5:**
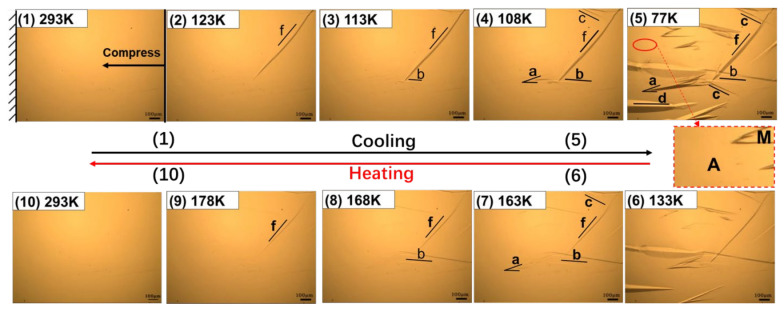
In situ observation of the thermoelastic martensitic transformation (1–5) and reverse transformation (6–10) with compressive stress. ‘A’ is austenite and ‘M’ is martensite.

The thermoelastic martensitic transformation during cooling and reverse transformation process during heating with compressive stress are shown in images 1–5 of Figure 5 and images 6–10 of Figure 5, respectively. The compressive stress was applied to the same sample as shown in Figure 4, and the schematic diagram is shown in Figure 3b,c. The martensitic transformation process under compressive stress was observed in the same field of view and with same experimental parameters as shown in Figure 4, and the other experimental parameters were also same as those in Figure 4.

As the temperature decreases, a martensite variant with a new orientation marked with “f” formed firstly, while the martensite with the orientation of “e” did not appear under the external compressive stress. The number of martensitic variants with orientations “a”, “b”, “c” and “d” decreased. The temperature corresponding to the initial appearance of the martensitic variants with each orientation also decreased. The order of appearance also changed to be “f” → “b” → “a” → “c” → “d”.

As shown in images 6–10 of Figure 5, the martensitic variants of five orientations (“f”,“b”, “a”, “c”, “d”) faded away, and the sample surface returned to its original smoothness again. The order of disappearance was “d” → “c” → “a” → “b” → “f”, which is also just opposite to the order of their formation. It is noted that the number of martensites with compressive stress was smaller than that without compressive stress.

### 3.3. Synchrotron Radiation X-ray Diffraction

**Figure 6:**
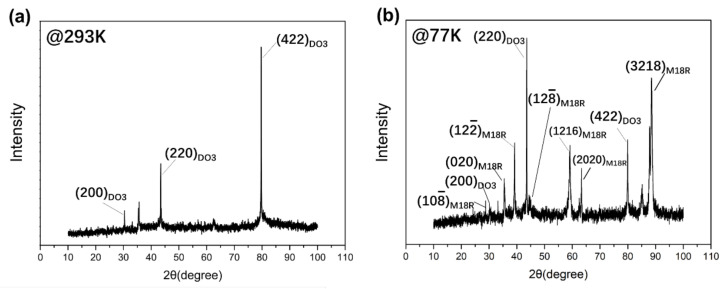
Synchrotron radiation X-ray diffraction spectrum of Cu-Al-Mn alloy at 293 K (**a**) and 77 K (**b**).

Figure 6 shows the synchrotron radiation X-ray diffraction spectrum of the same sample area of the Cu-Al-Mn alloy at 293 K and 77 K. As shown in Figure 6a, the parent phase of the Cu-Al-Mn cryogenic SMA at room temperature is AlCu_2_Mn phase, which is the DO_3_ structure. Figure 6b shows that there are $\left(10\bar{8}\right)$, (020), $\left(12\bar{2}\right)$, $\left(12\bar{8}\right)$, (1216), (2020) and (3218) diffraction peaks in the XRD pattern, which are unique to the M18R structure of martensite, and (200), (220) and (422) diffraction peaks of parent phase with DO_3_ structures.

According to the martensitic transformation temperature measurement results shown in Figure 1, the martensitic transformation should be completed at 77 K. However, the diffraction peaks of the parent/austenite phase are still there, as shown in Figure 6b, when the temperature is lower than Mf, which indicates that some parent/austenite phase remains in the sample after the martensitic transformation. The results of Figure 6b are consistent with the metallographic observation results of Figure 4 and Figure 5.

## 4. Discussion

During the process of thermoelastic martensitic transformation in SMA, it is assumed that there is a crystallographic relationship, “F”, between the martensite and the austenite: (h_1_k_1_l_1_)_M_//(h_2_k_2_l_2_)_P_, [u_1_v_1_w_1_]_M_//[u_2_v_2_w_2_]_P_. Based on this relationship, some martensites with crystallographically equivalent directions can be formed, which are named martensite variants. An austenite “A” with a certain crystallographic orientation would transform into many martensites, M_1_, M_2_, M_3_, …M_n_, during cooling. Here, we assumed that M_n_ is the last martensite during this transformation process. Then, all these martensites, M_1_, M_2_, M_3_, …M_n_, have to reverse to the same austenite “A” following the inverse relationship of relationship F, with the shape restoration of the SMA sample.

A question naturally arises. The martensite M_n_ may transform into any austenite A_i_ which has the crystallographically equivalent direction according to the relationship, “F”, during the reverse transformation process. However, the other austenite A_i_ may not keep the equivalence relation with the other martensite variants M_i_. This means that not all of the martensite variants transform into the same A_i_ following the relationship “F”. Another A_j_ must be formed, and the original shape of the materials cannot be restored. However, the restoration of the original shape of the materials (the parent phase) can always be observed in the experiment. It is suggested that the austenite can transform into different martensite variants during thermoelastic martensitic transformation, but all the martensite variants have to transform to the same original austenite during the reverse transformation. This is a kind of “asymmetry”. It is further speculated that the transformation from austenite to martensite cannot be completed to 100%, as a little parental austenite phase will remain. This is consistent with the current in situ experimental observations.

From the perspective of thermodynamics, the asymmetry between the transformation from austenite to martensite and its reverse transformation is possible. A Gibbs free energy change of a system upon the martensite transformation may be written as [27]:

(1) $$\Delta G^{P\to M}=\Delta G_{c}^{P\to M}+\Delta G_{i}^{P\to M}+\Delta G_{e}^{P\to M}$$

where $\Delta G_{c}^{P\to M}$ is a chemical energy term (with a negative value, the driving force for the transformation), $\Delta G_{i}^{P\to M}$ is an interface energy term (with a positive value, the resistance for the transformation) and $\Delta G_{e}^{P\to M}$ is an elastic strain energy term around the martensite (with a positive value, the resistance for the transformation). As a shear transformation, the martensitic transformation requires the adaptive deformation of austenite adjacent to martensite, which will produce an elastic strain energy $\Delta G_{e}^{P\to M}$. This elastic strain energy is the main resistance of the martensitic transformation as the low interface energy of the coherent interface between the martensite and the parent phase. During the martensitic transformation, the formation of multiple martensite variants can reduce the elastic strain energy by a self-coordinating effect.

During the reverse transformation, a Gibbs free energy change may be defined as Formula (2). If the reverse transformation is the growth process of residual austenites, this transformation process is the reverse shear process of the phase interface reverse migration (the disappearance order of variants in reverse transformation is opposite to that in martensitic transformation, which is the result of the reverse shear process). Martensitic transformation is a process of accumulating strain in the austenite phase, so the reverse transformation is a process of eliminating strain. This leads to an essential difference between Formula (2) and Formula (1); that is, the elastic strain energy term in Formula (2) will become negative and become the driving force of the phase transition. However, if the austenite phase is formed by nucleation in the martensite phase, this driving force is missing. Therefore, the growth of residual austenites has a driving force advantage over the nucleation of the austenite phase in the reverse transformation, which can also explain why it always changes back to the original austenite phase grain through the reverse transformation.

(2) $$\Delta G^{M\to P}=\Delta G_{c}^{M\to P}+\Delta G_{i}^{M\to P}+\Delta G_{e}^{M\to P}$$

Therefore, it is speculated that the growth of residual austenites will dominate the reverse transformation. Different martensitic variants transform into the original austenite grain, and in turn, the shape of the alloys is restored.

The direction of compressive stress applied to the Cu-Al-Mn SMA in this experiment is shown in Figure 3b. The relationship of the external stress (*F*) and the shear stress (*S*) in the martensite habit plane can be expressed as [28]:

(3) $$S=F\cdot \sin\left(\theta \right)\cdot \cos\left(\alpha \right)$$

The schematic diagram of force analysis is also shown in Figure 3c. The stress S will provide part of the driving force for the martensite transformation, which promotes the preferential formation of martensite with this orientation (such as f orientation here). While in the other nonequivalent direction, the compressive stress could hinder the formation of martensite, which will make the martensite variants with orientations of “a”, “b”, “c” and “d” appear later or even not appear (such as “e” orientation). With constant applied stress and heating, the order of disappearance of the thermoelastic martensitic variants in the inverse phase transition is just opposite to the order of their formation during in the martensitic phase transition. It is suggested that constant external stresses do not affect the release of the strain energy stored in the martensitic phase transition.

## 5. Conclusions

The thermoelastic martensitic transformation and its reverse transformation of the Cu-Al-Mn cryogenic SMA with and without compressive stress were dynamically in situ observed by using a self-designed cryogenic metallographic device. Some thermoelastic martensitic transformation rules were summarized. The size and number of martensites gradually increase up with the decrease of temperature, while they decrease with the increase of temperature, with and without the compressive stress. Different martensite variants grow continuously in the length and width directions. These preferentially grown martensite strips can interact with other oriented martensites subsequently formed. The order of martensite disappearance is just opposite to that of their formation. In the process of thermoelastic martensite transformation, even when the temperature is lower than M_f_, there are still some austenites which are not completely transformed into martensites. It means that the transformation from austenite to martensite was not completed to 100%. It is speculated that the martensite transformation of SMA would follow two stages, nucleation and growth, but its reverse transformation can occur only by the growth of residual austenites. If the reverse transformation can only occur by the growth of residual austenites, this can also explain why the deformed SMA remembers its original shape. The external compressive stress would change the appearance order of martensite variants, could induce new martensite variant and reduce some original martensite variants. Compared with the case without compressive stress, the growth of the original martensite variants could be restrained by the new stress field.

## Figures and Tables

**Table 1:** Chemical composition and Ms point of the sample.

Composition (wt.%)			Ms (K)
Cu	Al	Mn	
76.8	12.5	10.7	108

## Data Availability

The data can be made available from the corresponding author upon reasonable request.
